# Recommendations for Improving Ergonomics in Cleft Palate and Lip Surgery: A Scoping Review

**DOI:** 10.1177/22925503251386782

**Published:** 2025-10-24

**Authors:** Sara B. A. Morel, Rawan ElAbd, Youssef ElAbd, Dino Zammit, Sabrina Cugno

**Affiliations:** 1 Faculty of Medicine and Health Sciences, 12367 McGill University, Montreal, Quebec, Canada; 2 Department of Plastic, Reconstructive and Aesthetic Surgery, 5620 McGill University, Montreal, Quebec, Canada; 3 Faculty of Dentistry, 7938 University of Toronto, Toronto, Ontario, Canada

**Keywords:** Cleft palate, cleft lip, ergonomics, Ergonomie, fente labiale, fente palatine

## Abstract

**Introduction:** Craniofacial and cleft surgeries demand high precision, often leading to significant ergonomic challenges for surgeons. This review highlights critical recommendations for mitigating musculoskeletal disorders and enhancing surgeon well-being through equipment optimization, posture adjustments, and innovative technologies. **Methods:** To conduct this scoping review, a search of the literature yielded 18 studies which discuss ergonomics in the realm of cleft lip and palate surgery. **Results:** Eighteen studies were reviewed. Investigated ergonomic strategies in cleft lip and palate surgery encompassed headlights, loupes, microscopes, robots, videoscopes, endoscopes, exoscopes, positioning techniques, breaks, exercise, workshops, intubation methods, operating room design, and training programs. Most studies were classified as low risk of bias. Robotic systems and advanced visualization tools such as videoscopes and exoscopes demonstrated significant ergonomic benefits by improving surgeon posture, reducing musculoskeletal strain, and enhancing surgical precision. Microscopes and loupes offered improved magnification and comfort but were still associated with some reported musculoskeletal symptoms. Strategies including micro-breaks, exercise, ergonomic positioning, and optimized operating room setup showed potential to reduce physical discomfort during surgery. However, formal ergonomics education remains scarce in surgical training despite its recognized importance. **Conclusion:** Appropriate tools and positioning decrease the incidence of musculoskeletal disorders among cleft lip and palate surgeons. Further studies should focus on the outcomes of implementing such programs.

## Graphical abstract

**Graphical abstract:**
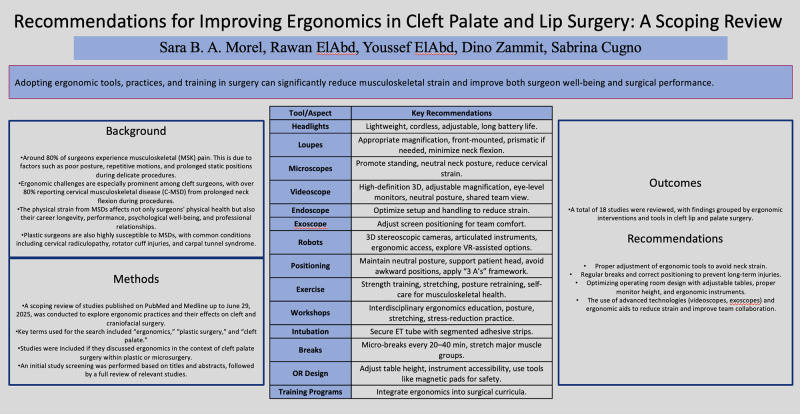
This is a visual representation of the abstract.

## Résumé

**Introduction : **La chirurgie craniofaciale et du palais demande une très grande précision, ce qui entraîne souvent des défis ergonomiques poru les chirurgiens. Cette revue souligne les recommandations essentielles visant à limiter les troubles musculo-squelettiques et à améliorer le bien-être des chirurgiens par une optimisation de l’équipement, un ajustement des postures et des technologies innovantes. **Méthodes : **Pour mener cette revue de portée, une recherche dans la littérature a fourni 18 études qui traitent de l’ergonomie dans le domaine de la chirurgie des fentes labio-palatines. **Résultats :** Dix-huit études ont été examinées. Les stratégies ergonomiques analysées dans la chirurgie des fentes labio-palatines portaient sur les lampes frontales, loupes, microscopes, robots, vidéoscopes, endoscopes, exoscopes, techniques de positionnement, interruptions et pauses, exercice, ateliers, méthodes d’intubation, conception de la salle d’opération et programmes de formation. La plupart des études étaient classées à faible risque de biais. Les systèmes robotiques et les outils de visualisation avancée, comme les vidéoscopes et exoscopes, démontraient des avantages ergonomiques significatifs en améliorant la posture du chirurgien, en réduisant la tension musculo-squelettique et en améliorant la précision chirurgicale. Les microscopes et loupes offraient un meilleur grossissement et confort, mais restaient associés au signalement de quelques symptômes musculo-squelettiques. Les stratégies incluant des micropauses, de l’exercice, un positionnement ergonomique et une installation optimisée de la salle d’opération mettaient en évidence un potentiel de réduction des désagréments physiques pendant l’intervention. Toutefois, une formation formelle en ergonomie reste rare dans la formation des chirurgiens, même si son importance est reconnue. **Conclusion :** Des outils et une position appropriés diminuent l’incidence des troubles musculo-squelettiques chez les chirurgiens opérant des fentes labio-palatines. Des études supplémentaires devraient porter sur les résultats de la mise en œuvre de tels programmes.

## Introduction

Occupational injuries are defined as damage from work-related events.^1^ Surgeons, particularly in demanding specialties like plastic surgery, face high risks of musculoskeletal disorders due to poor posture, repetitive motions, and prolonged static positions during delicate tasks.^2–4^

Around 80% of surgeons experience musculoskeletal (MSK) pain while operating, with over 30% sustaining occupational injuries and 9% ending their careers prematurely due to these issues.^5,6^ These injuries impair performance career longevity, psychological well-being, relationships, and sleep, increasing the risk of errors.^6–12^

Over 80% of cleft surgeons report cervical musculoskeletal disease (C-MSD) from prolonged neck flexion required for visualizing the palate.^2,13^ The small intraoral visual field further complicates visibility, limiting team interaction and learner participation.^14–16^

Surveys reveal that 64.2% of cleft surgeons experience MSK injuries, with neck (82%), lower back (56.6%), and shoulder (54.1%).^10^ These issues often lead to surgery (31%), sick leave (27%), or even early retirement (9.2%).^10,17^ Ergonomic practices, such as taking breaks, improving positioning, and mindfulness techniques, are essential for reducing these risks.^17^

Plastic surgeons are highly susceptible to MSDs, with 5% suffering from cervical radiculopathy and disc herniation and 4% of them undergoing discectomy and 2% requiring cervical fusion.^3^

Poor ergonomics, particularly sustained downward gaze during long surgeries, exacerbates cervical strain, especially when using loupes or headlamps.^18,19^ Other common MSDs include rotator cuff issues (18%) and carpal tunnel syndrome (9%), often caused by repetitive motions, forward head posture and static positions.^6,20^ This scoping review aims to elucidate different mechanism used to improve ergonomic in cleft surgery, providing new recommendations for reducing surgeon strain.

## Materials and Methods

This scoping review involved a comprehensive search of the literature in PubMed, Ovid, and CINAHL databases, from their inception up until June 29, 2025. The search used the following key terms: “cleft,” “cleft palate,” “cleft lip,” and “ergonomics.” The full search strategy is outlined in Supplementary Figure 1. To be eligible for inclusion, studies had to specifically address strategies and methods for improving ergonomics in cleft lip and palate surgery. The initial screening of titles and abstracts was carried out independently by two reviewers (S.B.AM and R.E.), with any disagreements resolved through discussion. Only full-text articles describing interventions related to surgical ergonomics were included in the analysis. Additionally, the references of all included articles were reviewed to identify further studies for possible inclusion.

Data extraction was performed using a standardized Microsoft Excel spreadsheet (Redmond, Washington, USA).^21^ The initial extraction was done by one reviewer (S.B.AM), with a second reviewer (R.E.) independently verifying the accuracy and completeness of the data. Statistical analysis was conducted on the compiled CLEFT patient data, with the different interventions summarized in the *Excel* document. This review was carried out in accordance with the Preferred Reporting Items for Systematic Reviews and Meta-Analysis (PRISMA)^22^ guidelines (Supplementary Figure 2).

## Risk of Bias Assessment

We performed a risk of bias (ROB) assessment for all studies included in our review. For observational cohort studies, we used the Newcastle-Ottawa scale (NOS),^23^ while the risk of bias in non-randomized studies of interventions (ROBINS-1 V2)^24^ was applied to non-randomized intervention studies.

## Results

A total of 18 studies were compiled into Table 1. Out of the studies, 2 were non-randomized observational studies,^25,26^ 2 were retrospective cohort studies,^10,27^ and 14 were prospective cohort studies.^16,17,28–39^ The number of studies investigating each ergonomic strategy for cleft surgery is as follows: headlights: 2^10,17^; loupes: 3^10,17,32^; microscope: 3^10,25,26^; robot: 6^27,29,33,35,36,38^; videoscope: 4;^16,28,37,39^ endoscope: 1^39^; exoscope: 3^31,34,39^; head/neck positioning: 2^10,17^; breaks: 1^17^; exercise: 2^10,17^; workshops: 1^17^; intubation: 1^30^; OR design: 1^17^; and training programs: 2.^10,17^ A summary of the findings for each recommendation category can be found in Table 2.

**Table 1.** Overview of 18 Studies Included in Review.

Authors	Publication Year	Findings
Meier et al	2022	Videoscopes: The *Vitom 3D^®^* microvideoscope enhances ergonomics by enabling a seated, neutral head posture and reducing neck and back strain, while offering advanced features like 4K imaging, 3D visualization, and shared views for the surgical team.
Smartt et al	2012	Robots: The *da Vinci Surgical System* has shown significant advantages in cleft surgeries, providing superior visibility, exposure, and reduced surgeon fatigue through its robotic arms and 30-degree camera.
Bajaj et al	2012	Intubation: The innovative endotracheal tube fixation technique using adhesive *Elastoplasts* provides greater stability and reliability.
Hamdan et al	2024	Headlights: Lightweight, cordless headlights with adjustable headbands, long-lasting batteries, and customizable light intensity improve surgeon visibility, reduce eye strain, and enhance comfort during cleft lip and palate surgeries.
		Loupes: Properly selected front-mounted loupes with appropriate magnification, optical quality, and ergonomic features improve surgical precision, reduce eye fatigue, and promote better posture and comfort during prolonged cleft surgeries.
		Positioning: The “3 A's” framework, Avoid, Adopt, and Advance, emphasizes the importance of ergonomic practices in cleft surgery, promoting neutral posture, correct arm and wrist alignment, and patient positioning with supportive headrests to reduce strain and improve focus, ultimately enhancing surgical outcomes.
		Exercise: Ergonomic interventions like strength training, exercise, and posture retraining are crucial for reducing pain and improving surgical performance, but time constraints and inconsistent adherence remain significant barriers to their effectiveness, as many surgeons rely on self-treatment methods like stretching and over-the-counter medications to manage musculoskeletal discomfort.
		Workshops: The Ergonomics and Musculoskeletal Wellness Workshop, part of the Comprehensive Cleft Care Workshop, effectively raises awareness about posture and injury prevention through collaborative sessions and practical techniques like yoga and stretching.
		Breaks: Micro-breaks, lasting 40 to 120 s every 20 to 40 min, help reduce muscle fatigue and physical discomfort during long procedures by allowing surgeons to stretch key muscle groups, although challenges in maintaining sterility and disrupting surgical flow may limit their application.
		Operating Room Design: Optimizing operating room setup for cleft surgery by adjusting the surgical table height to align with the surgeon's neutral posture and ensuring efficient instrument layout can reduce strain and improve comfort, while using a *Magnetic Instrument Pad* enhances safety by minimizing the risk of accidental injury during instrument passing.
		Training Programs: The lack of formal ergonomics training in surgical programs contributes to surgeon well-being and performance issues, with a significant gap in education despite advanced specializations, as many surgeons rely on self-treatment for physical strain instead of professional care.
Chear Lee et al	2025	Exoscopes: The *VITOM^®^ 3D* exoscope improved ergonomics in cleft surgeries by reducing neck flexion and time spent in uncomfortable postures, while offering superior image quality, magnification, and illumination, though some assistants reported minor discomfort from screen positioning.
Kuang et al	2017	Loupes: Prismatic glasses that refract the visual field by 90° significantly reduce neck and back discomfort and time spent in neck-flexed positions during cleft surgery, improving surgeon comfort without affecting surgical duration or outcomes.
Podolsky et al	2016	Robots: The *da Vinci Xi* system offered superior wrist articulation, reachability, and fewer instrument collisions than the *Si* system, though both robotic systems resulted in longer operative times compared to conventional techniques.
Chebib et al	2022	Exoscopes: The *VITOM^®^ 3D* exoscope has 4 K 3D imaging, ease of setup, and enhanced team collaboration, with surgeons reporting increased comfort and efficiency as they gained experience.
Tanaka et al	2020	Microscopes: Compared to the traditional *Rose* position, using an operating microscope with a standing posture reduces cervical strain by promoting a neutral neck position, improving comfort, visibility, and ergonomic benefits for the entire surgical team.
Mokhtar et al	2025	Robots: The *RoboticScope* improved visualization, precision, and ergonomics in pediatric cleft repair, though it resulted in longer surgery times and higher costs, while also reducing postoperative opioid use and complications compared to conventional methods.
Nadjmi et al	2016	Robots: The *da Vinci* robot improved dexterity, precision, and depth perception during palatal muscle dissection and suturing, resulting in better ergonomics and shorter hospitalization, although robotic-assisted procedures took longer.
Nguyen et al	2016	Robots: Robotic-assisted cleft repair and radical intravelar-veloplasty showed comparable dissection times to manual approaches, with improved levator repair and oral mucosa closure times as surgeons gained experience, all while maintaining excellent visibility and ergonomic comfort.
Sommerlad	2003	Microscopes: The *Zeiss OpMi Vario* operating microscope offers superior magnification and illumination with adjustable focal length, significantly reducing neck flexion and spine strain during cleft palate repair while enhancing surgical precision and educational opportunities despite a learning curve and sterility challenges.
Allori et al	2014	Videoscopes: The integration of video-assisted technology into traditional palatopharyngeal surgery improves visibility, ergonomics, and education by providing high-definition real-time visualization, reducing reliance on headlamps, eliminating the need for surgeons to bend over the operative field, and enhancing trainee learning through shared video footage.
Levites et al	2020	Videoscopes: The videoscope system for primary cleft repair enhances ergonomics by reducing neck strain, improves visibility for both surgeons and the team, and supports resident learning and autonomy.
Shah et al	2021	Headlights: Although headlamps are widely used to aid visualization during craniofacial procedures, their use, especially combined with loupes or microscopes, is associated with increased musculoskeletal pain.
		Loupes: While loupes are widely used, their use is associated with higher musculoskeletal pain, especially in more experienced surgeons, though prismatic loupes may help reduce discomfort and improve posture.
		Microscopes: Despite the ergonomic benefits of microscopes, surgeons still experience higher levels of musculoskeletal pain, particularly in the neck and upper body.
		Positioning: Ergonomic interventions like strength training, exercise, and posture retraining can reduce pain and improve surgical performance.
Khan et al	2015	Robots: The *da Vinci Si* Surgical Robot improved ergonomics, visibility, and maneuverability in trans-oral cleft surgery by using smaller instruments and enhancing access to the posterior pharynx and palate.
Twefik et al	2024	Endoscopes: The exoscope was preferred over the endoscope in cleft surgery due to its extraoral positioning, higher magnification, and improved ergonomics, offering better handling, image quality, and reduced physical strain, while maintaining similar execution times but with more efficient setup.
		Exoscopes: Exoscopes offer significant ergonomic benefits in cleft surgery by minimizing intraoral clutter, improving surgeon posture, and enhancing visualization with greater magnification and 3D imaging.

**Table 2.** Summary of Recommendations.

Tool/Aspect	Recommendations
Headlights	Use lightweight, cordless headlights to minimize fatigue during prolonged procedures. Choose headlights with adjustable, comfortable headbands to ensure a secure fit without excessive pressure. Opt for models with long-lasting batteries to avoid interruptions and maintain consistent illumination.
Loupes	Select loupes with appropriate magnification balancing surgical precision and comfort. Choose front-mounted loupes featuring clear lenses, low-set frames, and steep declination angles to reduce eye fatigue and promote natural head posture. Consider prismatic loupes or prismatic glasses to reduce neck and back strain by minimizing neck flexion without compromising surgical performance.
Microscopes	Adopt standing postures facilitated by microscope use to maintain a neutral neck position and reduce cervical strain compared to traditional positions requiring loupes and headlights.
Videoscope	Use videoscopes with high-definition, 3D visualization and adjustable magnification to enhance surgical precision while supporting ergonomic posture. Position the surgeon seated with a neutral head and neck posture, reducing strain on the neck and back during procedures. Employ lightweight 3D glasses compatible with prescription lenses and maintain a comfortable viewing distance to minimize discomfort. Utilize video-assisted systems that allow the surgical team to share a clear, magnified view, improving teamwork and educational opportunities. Leverage multiple monitor setups, including eye-level displays, to reduce neck flexion and promote neutral posture for both surgeons and assistants.
Endoscope	Optimize setup and handling techniques to minimize discomfort related to restricted movement and positioning challenges.
Exoscope	Monitor assistant comfort and adjust screen positioning as needed to minimize minor ergonomic discomfort for the surgical team.
Robots	Employ robotic systems with 3D stereoscopic cameras and articulated instruments (eg, da Vinci) to enhance visualization, dexterity, and precision during delicate cleft procedures. Take advantage of robotic wrist articulation and improved reach to access difficult anatomical areas while minimizing surgeon strain. Explore emerging technologies like virtual reality-controlled microscopes (eg, RoboticScope) for further ergonomic and visualization benefits, while accounting for learning curves and device-specific discomforts.
Positioning	Maintain a neutral surgeon posture with proper alignment of the head, neck, arms, and wrists to minimize musculoskeletal strain. Use supportive devices such as silicone gel donut headrests to stabilize the patient's head and optimize surgical field visualization. Avoid sustained awkward or uncomfortable postures by adjusting both surgeon and patient positioning throughout the procedure. Incorporate the “3 A's” framework: Avoid harmful postures, Adopt ergonomic positioning techniques, and Advance habits through regular ergonomic exercises. Engage in strength training, stretching, and posture retraining outside the operating room to enhance endurance and reduce pain during surgery.
Exercise	Incorporate regular strength training and stretching routines to build musculoskeletal endurance and reduce the risk of injury. Practice posture retraining exercises to reinforce proper alignment during surgery and daily activities. Use self-care strategies such as targeted stretching and physical therapy to manage and prevent musculoskeletal discomfort.
Workshops	Implement interdisciplinary ergonomics workshops that educate surgeons and care providers on musculoskeletal wellness and injury prevention. Include practical sessions on posture awareness, stretching, strengthening exercises, and stress-reduction techniques like yoga.
Intubation	Use adhesive *Elastoplast* strips cut into three segments to secure the endotracheal tube: one central strip on the lower lip and two lateral strips extending below the lip angles.
Breaks	Incorporate micro-breaks lasting 40 to 120 s every 20 to 40 min during surgery. Use breaks to stretch key muscle groups: neck, shoulders, back, wrists, hands, knees, and ankles. Plan breaks collaboratively with the surgical team to minimize impact on procedure continuity.
Operating Room Design	Adjust the surgical table height to the surgeon's neutral posture, typically between 66 to 77 cm from the floor, aligning with elbow or waist level. Ensure the table height promotes comfort and minimizes strain on the surgeon's neck, shoulders, and back. Organize instruments for easy and efficient accessibility within the surgeon's immediate reach. Utilize tools like Magnetic Instrument Pads to securely hold instruments and reduce the risk of accidental injury during passing.
Training Programs	Integrate formal ergonomics education into surgical training curricula to address current gaps. Teach ergonomic principles and proper use of equipment to improve surgeon well-being and performance.

## Risk of Bias Assessment

As outlined in Table 3, The ROBINS-1 V2 tool was applied to 2 studies,^25,26^ while the Newcastle-Ottawa Scale (NOS) was used in 16 studies.^10,16,17,27–39^ Overall, 88.89% (n = 16) of the studies were classified as low risk, while 11.11% (n = 2) were classified as moderate risk. The higher risk studies were primarily due to the absence of a comparison group.

**Table 3.** Risk of Bias Assessment

Observational Studies: Newcastle-Ottawa Scale (NOS)														
**Author**	**Year**	**1) Representativeness of the exposed cohort** **a) truly representative of the average _______________ (describe) in the community** **b) somewhat representative of the average ______________ in the community** **c) selected group of users e.g., nurses, volunteers** **d) no description of the derivation of the cohort**	**2) Selection of the non-exposed cohort** **a) drawn from the same community as the exposed cohort** **b) drawn from a different source** **c) no description of the derivation of the non- exposed cohort**	**3) Ascertainment of exposure** **a) secure record (e.g., surgical records)** **b) structured interview** **c) written self -eport** **d) no description**	**4) Demonstration that outcome of interest was not present at start of study** **a) yes** **b) no**	**Total Selection Stars**	**1) Comparability of cohorts on the basis of the design or analysis** **a) study controls for _________ (select the most important factor)** **b) study controls for any additional factor (This criterion could be modified to indicate specific control for a second important factor.)**	**Total Comparability Stars**	**1) Assessment of outcome** **a) independent blind assessment** **b) record linkage** **c) self report** **d) no description**	**2) Was follow-up long enough for outcomes to occur** **a) yes (select an adequate follow up period for outcome of interest)** **b) no**	**3) Adequacy of follow up of cohorts** **a) complete follow up - all subjects accounted for ¯** **b) subjects lost to follow up unlikely to introduce bias - small number lost - > ____ % (select an adequate %) follow up, or description provided of those lost) ¯** **c) follow up rate < ____% (select an adequate %) and no description of those lost** **d) no statement**	**Total Outcome Stars**	**Total Stars**	**Overall Risk**
**Meier et al**	**2022**	a: Undergoing cleft palate repairs in the community	c	a	a	3	a: Age b: Gender, Comorbidities, Surgical technique	2	a	b	d	1	6	Moderate
**Smartt et al**	**2012**	a: Cadaveric human heads in the community	d	a	a	3	a: Robot system	1	b	a	a	3	7	Low
**Bajaj et al**	**2012**	a: Patients undergoing palate surgery) in the community	a	a	a	4	a: Age b: Gender	2	a	a	a	3	9	Low
**Hamdan et al**	**2024**	a: Cleft care providers in the community	c	a	a	3	a: Ergonomic knowledge of providers	1	a	a	a	3	7	Low
**Chear Lee et al**	**2025**	a: Pediatric cleft surgeons and surgical staff in the community	a	a	a	4	a: Neck position of surgeons and assistants	1	a	a	a	3	8	Low
**Kuang et al**	**2017**	a: Surgeons in the community	a	a	a	4	a: Surgeons’ experience	1	a	a	a	3	8	Low
**Podolsky et al**	**2016**	a: Surgeons in the community	c	a	a	3	a: Surgeons’ experience b: Robotic system type	2	a	a	a	3	8	Low
**Chebib et al**	**2022**	a: Children undergoing pediatric cleft palate surgeries in the community	a	a	a	4	a: Type of surgery	1	a	a	a	3	8	Low
**Mokhtar et al**	**2025**	a: Pediatric patients with isolated, nonsyndromic cleft palate in the community	b	a	a	3	a: Cleft type	1	a	a	b	3	7	Low
**Nadjmi et al**	**2016**	a: Cadaveric human heads in the community	b	a	a	3	a: Hand dissection	1	a	b	b	3	7	Low
**Nguyen et al**	**2016**	a: Cadaveric human heads in the community	b	a	a	3	a: Hand dissection	1	a	b	b	3	7	Low
**Allori et al**	**2014**	a: Trainee in the community (Residents, students, and junior residents involved in cleft palate surgery training)	a	a	a	4	a: Surgical training experience	1	a	a	b	2	7	Low
**Levites et al**	**2020**	a: Patients in the community	a	a	a	4	a: Procedure type	1	a	a	a	3	8	Low
**Shah et al**	**2021**	a: Surgeon practicing craniofacial surgery in the community	b	c	b	2	a: Gender b: Years of practice	2	b	b	c: < 80%	2	6	Moderate
**Khan et al**	**2015**	a: Cadaveric human heads in the community	b	a	a	3	a: Hand dissection	1	a	b	b	3	7	Low
**Twefik et al**	**2024**	a: Beginner surgeons in the community	a	a	a	4	a: Experience in transoral surgery	1	a	a	a	3	8	Low

Non-Randomized Studies of Interventions: ROBINS-I V2		
**Author**	**Tanaka**	**Sommerlad**
**Year**	2020	2003
**Did the authors make any attempt to control for confounding?**	Yes	Yes
**Is there sufficient potential for confounding that an unadjusted result should not be considered further?**	NA	NA
**Was the method of measuring the outcome inappropriate?**	No	No
**Did the authors control for all the important confounding factors for which this was necessary?**	Yes	Yes
**Were confounding factors that were controlled for (and for which control was necessary) measured validly and reliably by the variables available in this study?**	Yes	Yes
**Did the authors control for any post-intervention variables that could have been affected by the intervention?**	Yes	Yes
**Did the use of negative controls, quantitative bias analysis, or other considerations, suggest serious unmeasured confounding?**	No	No
**Risk of Bias**	Low	Low
**Did the authors use an analysis method that was appropriate to control for time-varying as well as baseline confounding?**	Yes	Yes
**Did the authors control for all the important baseline and time-varying confounding factors for which this was necessary?**	Yes	Yes
**Were confounding factors that were controlled for (and for which control was necessary) measured validly and reliably by the variables available in this study?**	Yes	Yes
**Did the authors control for time-varying factors or other variables measured after the start of intervention?**	NA	NA
**Did the use of negative controls, or other considerations, suggest serious unmeasured confounding?**	No	No
**Risk of Bias**	Low	Low
**Did assignment of participants to the intervention group or the comparator group rely on events or measurements that occurred after the start of follow-up?**	No	No
**Were participants included in the comparator group until they fulfilled the definition of the intervention (or vice versa)?**	NA	NA
**Was all information used to classify intervention and comparator groups recorded at or before the time the interventions started?**	Yes	Yes
**Was classification of intervention status influenced by knowledge of the outcome or risk of the outcome?**	No	No
**Was intervention status classified correctly for all, or nearly all, participants?**	NA	NA
**Risk of Bias**	Low	Low
**Did assignment of participants to the intervention group or the comparator group rely on events or measurements that occurred after the start of follow-up?**	No	No
**Were participants excluded after the start of follow-up because they did not meet the definition of either the intervention or the comparator?**	NA	NA
**Were start of follow-up and start of intervention the same for most participants?**	Yes	Yes
**Is the effect of intervention expected to be constant over the time period studied?**	Low	Low
**Was selection of participants into the study (or into the analysis) based on participant characteristics observed after the start of intervention (additional to the situations addressed in 3.1 and 3.3)?**	No	No
**Were the post-intervention variables that influenced selection likely to be associated with intervention?**	NA	NA
**Were the post-intervention variables that influenced selection likely to be influenced by the outcome or a cause of the outcome?**	NA	NA
**Is it likely that the analysis corrected for all of the potential selection biases identified in 3.1–3.2, 3.3–3.4 or 3.5–3.7 above?**	NA	NA
**Did sensitivity analyses demonstrate that the likely impact of the potential selection biases identified in 3.1–3.2, 3.3–3.4 or 3.5–3.7 above was minimal?**	NA	NA
**Were potential selection biases identified in 3.1–3.2, 3.3–3.4 or 3.5–3.7 above sufficiently severe that the result should not be included in a quantitative synthesis?**	NA	NA
**Risk of Bias**	Low	Low
**Was the study undertaken in an experimental context?**	No	No
**Did participants deviate from the intended intervention as a result of the processes of recruiting and engaging them in the study?**	NA	NA
**Did study personnel consciously or unconsciously undermine implementation of the intended interventions?**	No	No
**Were these deviations from intended intervention likely to have affected the outcome?**	No	No
**Was an appropriate analysis used to estimate the effect of assignment to intervention?**	NA	NA
**Risk of Bias**	Low	Low
**Did all or nearly all participants adhere to their assigned intervention strategy?**	Yes	Yes
**Were the protocol deviations likely to have affected the outcome?**	No	No
**Was an appropriate analysis used to estimate the specified per-protocol effect, accounting for the specified protocol deviations?**	NA	NA
**Risk of Bias**	Low	Low
**Were complete data on intervention status available for all, or nearly all, participants?**	Yes	Yes
**Were complete data on the outcome available for all, or nearly all, participants?**	Yes	Yes
**Were complete data on important confounding variables available for all, or nearly all, participants?**	Yes	Yes
**Is the result based on a complete case analysis?**	NA	NA
**Was exclusion from the analysis because of missing data (in intervention, confounders, or the outcome) likely to be related to the true value of the outcome?**	No	No
**Is the relationship between the outcome and missingness likely to be explained by the variables in the analysis model?**	Yes	Yes
**Was the analysis based on imputing missing values?**	NA	NA
**Is it reasonable to assume that data were ‘missing at random’ (MAR) or ‘missing completely at random’ (MCAR)?**	Yes	Yes
**Was imputation performed appropriately?**	No	No
**Was an appropriate alternative method used to correct for bias due to missing data?**	NA	NA
**Is there evidence that the result was not biased by missing data?**	Yes	Yes
**Risk of Bias**	Low	Low
**Could measurement or ascertainment of the outcome have differed between intervention groups?**	No	No
**Were outcome assessors aware of the intervention received by study participants?**	NA	NA
**Could assessment of the outcome have been influenced by knowledge of the intervention received?**	No	No
**Risk of Bias**	Low	Low
**Was the result reported in accordance with an available, pre-determined analysis plan?**	Yes	Yes
**Is the numerical result being assessed likely to have been selected, on the basis of the results, from multiple outcome measurements (eg, scales, definitions, time points) within the outcome domain?**	No	No
**Is the numerical result being assessed likely to have been selected, on the basis of the results, from multiple analyses of the data?**	No	No
**Is the numerical result being assessed likely to have been selected, on the basis of the results, from multiple subgroups?**	No	No
**Risk of bias**	Low	Low
**Overall risk of bias**	Low	Low

## Discussion

### Ergonomic Recommendations for Surgeons

#### Headlights

Hamdan^17^ evaluated ergonomic strategies aimed at enhancing surgeon comfort and efficiency during cleft lip and palate surgeries. A central recommendation was the use of lightweight, cordless headlights equipped with adjustable headbands, long-lasting batteries, and customizable light intensity. These features collectively enhance visibility, reduce eye strain, and improve overall comfort.

Shah^10^ investigated MSK symptoms and ergonomic practices among members of the American Cleft Palate-Craniofacial Association. In a survey of 873 surgeons, 79.5% reported using headlamps during craniofacial procedures. While these devices aid in visualization, their use, particularly when combined with loupes or microscopes, was associated with higher reported pain scores.

### Loupes

Hamdan^17^ emphasized the critical role of properly selected loupes in cleft lip and palate surgery, highlighting the importance of magnification strength, optical quality, and ergonomic design. Front-mounted loupes with 2.5×, 3.5×, or 4.5× magnification were recommended for their ability to enhance surgical precision while maintaining comfort. Key ergonomic features, such as clear lenses, low-set frames, and steep declination angles, not only improve visualization but also help reduce eye fatigue. These loupes promote a wider working distance, which facilitates better arm positioning and contributes to sustained ergonomic posture during complex, prolonged procedures.

In a broader survey, Shah^10^ found that 83.2% of surgeons used loupes in practice. However, those who did report higher pain scores compared to users of headlights or microscopes. Notably, surgeons with over 15 years of experience had nearly twice the odds of reporting MSK symptoms, underscoring the long-term physical toll of poor ergonomic practices.

Supporting these findings, Kuang^32^ examined the use of prismatic glasses, which refract the visual field by 90°, in cleft palate surgeries performed by oral-maxillofacial surgeons. In a controlled comparison involving three surgeons and six patients, the use of prismatic glasses led to significantly reduced neck and back discomfort, along with less time spent in neck-flexed positions. Importantly, these ergonomic benefits were achieved without compromising surgical duration or outcomes.

### Microscopes

Sommerlad^26^ explored the use of the *Zeiss OpMi Vario*^40^ operating microscope in cleft palate repair, highlighting its advanced features such as a tiltable binocular, beam splitter, teaching arm, and integrated video camera. This microscope provides superior magnification and illumination compared to traditional loupes or headlights, while its adjustable focal length allows for precise visualization. Critically, it supports improved neck ergonomics by minimizing the flexion required during prolonged procedures. The system also enhances the educational experience, enabling assistants and trainees to follow the procedure via the teaching arm or video monitor. While there is a learning curve and initial manual adjustments can be time-consuming, the ergonomic and visual benefits, along with improved surgical precision, make it a valuable asset.

Tanaka^25^ compared the traditional *Rose* position with a standing posture facilitated by the operating microscope in cleft surgery. The *Rose* position, which involves considerable neck flexion to accommodate loupes and headlights, was associated with increased cervical strain. In contrast, the standing posture allowed by the microscope helped maintain a neutral neck position, improving comfort and reducing MSK stress for both surgeons and assistants. The microscope also promoted visibility and posture, regardless of height. While the setup requires specialized training and may not be feasible in all resource settings, its benefits in ergonomics were evident.

However, Shah^10^ reported that despite these advantages, surgeons using microscopes still experienced higher levels of MSK pain, particularly in the neck and upper body. These findings underscore the importance of proper ergonomic training and techniques, even when using tools designed to improve posture and visualization.

### Videoscope

Meier^28^ evaluated the *Vitom 3D*^®^(StorzEndoscope) microvideoscope across eight consecutive cleft palate repairs, highlighting its advanced features such as 4K imaging, 3D visualization, and 30× magnification, all controlled via a sterile joystick. This system improved surgeon ergonomics by enabling a seated position with a neutral head posture, significantly reducing neck and back strain. Lightweight 3D glasses, compatible with prescription lenses, and a comfortable 2-m viewing distance further minimized discomfort. Additional benefits included enhanced image clarity, precise surgical performance, and a shared 3D view for the entire surgical team, which supported teamwork and education.

Allori^37^ examined the integration of video-assisted technology into traditional palatopharyngeal surgery to enhance visibility, ergonomics, and education. Maintaining the conventional position with the surgeon at the patient's head, the setup incorporated a high-definition laparoscope connected to a video tower for real-time visualization. Using a flexible *Endocameleon* scope^41^ positioned upside down to match the surgeon's perspective, this approach optimized lighting and oropharyngeal views while allowing smooth transitions between conventional and video-assisted methods. The video footage was recorded to create an educational library for asynchronous learning. Benefits included improved visualization and better ergonomics by eliminating the need to bend over the operative field.

Levites^16^ investigated a videoscope system for cleft repair featuring a 5-mm, 30-degree high-definition laparoscope mounted on a holding arm. Surgeons could alternate between direct and video-assisted views, with images displayed on multiple monitors, including one positioned at eye level. This setup improved ergonomics by reducing neck strain and enhanced visibility for both surgeons and the surgical team. Despite greater resident involvement, operative times remained comparable. Advantages included increased surgeon comfort, superior visualization, enriched resident learning, and real-time monitoring via multiple room monitors that allowed residents greater autonomy while enabling detailed feedback from attending surgeons. Additionally, the system improved operating room efficiency and generated an educational video archive. The primary drawbacks were the need for additional equipment and setup time.

### Endoscope

Twefik^39^ compared endoscopes and exoscopes in cleft surgery. Execution times were similar, but the exoscope had a clear advantage in setup. Feedback from questionnaires showed that the exoscope was less demanding, with higher satisfaction ratings for setting, handling, image quality, and 3D capabilities. Both devices improved ergonomics by reducing physical strain, but the exoscope was preferred due to its extraoral positioning, higher magnification, and reduced intraoral encumbrance. Endoscopes are commonly used in cleft surgeries for their compact size and high-quality 2D/3D images, but their rigid positioning and intraoral placement can limit mobility.

### Exoscopes

Exoscopes provide notable ergonomic advantages over traditional endoscopes in cleft surgery. Their extraoral placement minimizes intraoral clutter, allowing surgeons to maintain a more natural and comfortable posture. Additionally, the enhanced magnification and 3D imaging capabilities of exoscopes improve visualization of critical anatomical structures, which is essential for successful repairs.^39^

Chear Lee^31^ investigated the ergonomic benefits and surgical outcomes of the *Vitom3D*^®^exoscope in pediatric cleft surgeries, comparing its performance to conventional tools such as loupes and microscopes. The study measured surgeons’ neck flexion angles during procedures. Use of the *Vitom3D*^®^exoscope significantly reduced the time spent in ergonomically unfavorable postures (less than 10% compared to 93% with traditional loupes) and enhanced overall working ergonomics. Surgeons rated the exoscope highly for image quality, magnification, illumination, and ergonomic comfort, although some assistants reported minor discomfort related to screen positioning.

Similarly, Chebib^34^ evaluated the *Vitom3D*^®^exoscope across a range of pediatric surgeries, including cleft repairs. Surgeons compared the exoscope to microscopes and loupes on factors such as ease of setup, visualization, lighting, and comfort. The exoscope was strongly preferred, with 94%–97% of surgeons rating it superior in all categories. Its 4 K 3D imaging and screen-based visualization not only enhanced surgical precision but also improved collaboration and communication among team members by allowing everyone to view the procedure on the same display. As surgeons gained experience with the device, they reported increased comfort and efficiency.

### Robots

Robotic systems, particularly the *da Vinci Surgical System,*^42^ have demonstrated significant promise in improving both precision and ergonomics in cleft palate and lip surgeries. Smartt^29^ conducted cadaveric studies using the da Vinci system for posterior pharyngeal flap surgery, finding that the robotic arms and 30-degree camera offered superior visibility and exposure compared to traditional methods, while reducing surgeon fatigue. This early feasibility was expanded upon by Podolsky,^33^ who used a high-fidelity simulator based on infant CT data to compare the *da Vinci Si* and *Xi* systems. The *Xi* system outperformed the *Si* with better wrist articulation, reachability, and fewer instrument collisions, although both systems resulted in longer operative times than conventional techniques. Further clinical application was demonstrated by Nadjmi,^35^ who utilized the *da Vinci* robot for palatal muscle dissection in cadaver trials and then in 10 pediatric patients. The robot's 3D stereoscopic camera and *EndoWrist* instruments enhanced dexterity, precision, and depth perception, improving visibility during dissection and suturing. While robotic-assisted procedures averaged 122 min, longer than the 87 min of manual surgery, they provided better ergonomics and shortened hospitalization from 2.4 to 1 day. Similarly, Nguyen^36^ investigated robotic-assisted cleft repair and radical intravelar-veloplasty in cadaveric heads, demonstrating comparable dissection times between robotic and manual approaches. With experience, surgeons improved levator repair and oral mucosa closure times while maintaining excellent visibility and ergonomic comfort. Khan^38^ further explored the *da Vinci Si Surgical Robot*'s feasibility for trans-oral cleft surgery in a pediatric airway manikin, testing various configurations to optimize access and safety.

More recently, Mokhtar^27^ introduced the *RoboticScope*,^43^ a 3D virtual reality microscope controlled via head gestures. Compared to conventional surgery, the *RoboticScope* improved visualization, precision, and ergonomics, though surgeries took on average 28 min longer. Importantly, patients in the robotic group required less postoperative opioid use and experienced no complications, contrasting with one uvula dehiscence in the conventional cohort. Despite its benefits, the *RoboticScope* high cost, learning curve, and potential discomfort from the head-mounted display pose limitations.

### Positioning

Hamdan^17^ emphasizes the critical role of ergonomic practices in healthcare, particularly in cleft surgery, introducing the *3 A's framework*: Avoid, Adopt, and Advance. This approach encourages surgeons to avoid uncomfortable postures, adopt proper positioning techniques, and advance their ergonomic habits by incorporating exercises into their routines. In cleft palate surgery specifically, maintaining a neutral posture with correct arm and wrist alignment is essential for reducing strain and enhancing focus. Patient positioning is equally important, with the use of a silicone gel donut headrest to support the head and optimize surgical visualization.

Supporting this, Shah^10^ highlights the value of ergonomic interventions such as strength training, exercise, and posture retraining to reduce pain and improve surgical performance over the long term. However, Shah also notes challenges surgeons face in committing time to these practices and sustaining ideal posture during lengthy procedures.

### Exercise

Hamdan^17^ found that ergonomic interventions, including strength training, exercise, and posture retraining, play a vital role in reducing pain, enhancing surgical performance, and supporting long-term health among surgeons. Complementing this, Shah^10^ reported that surgeons commonly use self-treatment methods such as stretching, over-the-counter medications, and strength training to manage MSK discomfort. Treatments often involve physical therapy and diagnostic imaging. Shah's study highlighted that MSK issues frequently impair posture, stamina, and surgical speed, with nearly 30% of surgeons requiring prescription medications or injections. However, time constraints and inconsistent adherence remain significant barriers to the effectiveness of these interventions.

### Workshops

This study by Hamdan^17^ describes the *Comprehensive Cleft Care Workshop*, an annual interdisciplinary event that equips cleft care providers with evidence-based knowledge and technical skills. A key component, the *Ergonomics and Musculoskeletal Wellness Workshop*, addresses MSK challenges faced by providers through collaborative sessions led by surgeons, dentists, therapists, and psychologists. The workshop introduces ergonomic principles and practical techniques such as yoga, stretching, and strengthening exercises to promote long-term health and reduce injury risk. While the session effectively raises awareness about posture, technology use, and injury prevention, its brief two-hour duration may limit skill retention.

### Intubation

Bajaj^30^ presents an innovative method for endotracheal tube fixation during cleft lip and palate surgery using adhesive *Elastoplasts,*^44^ cut into three segments, a 2 cm central strip fixed to the lower lip and two 1.5 cm lateral strips extending below the lip angles. This technique stabilizes the tube, prevents lateral movement, and eases mouth gag application, offering greater reliability than single-point adhesive methods.

### Breaks

Hamdan^17^ examined the impact of micro-breaks, lasting 40 to 120 s every 20 to 40 min. These breaks allowed surgeons to stretch key muscle groups, including the neck, shoulders, back, wrists, hands, knees, and ankles, while maintaining sterility. The practice was found to improve ergonomics by reducing muscle fatigue and physical discomfort during long procedures. However, potential drawbacks include the risk of disrupting surgical flow.

### Operating Room Design

Hamdan^17^ explored the ergonomic optimization of operating room setup for cleft surgery, focusing on surgical table height and instrument accessibility. It recommends adjusting the table to the surgeon's neutral posture, typically 66 to 77 cm from the floor, aligning with elbow or waist level, to promote comfort and reduce strain. The study also highlights the importance of efficient instrument layout and the use of a magnetic instrument pad to enhance safety by minimizing the risk of accidental injury.

### Training Programs

Hamdan^17^ highlights a gap in surgical education, noting the lack of formal ergonomics training. Incorporating ergonomic principles and equipment into training curricula could improve surgeon well-being and performance, while reducing the risk of MSK injuries. Supporting this, Shah^10^ found that despite 71% of fellowship-trained surgeons having advanced specializations, only 7.4% reported formal ergonomics education. This significant shortfall reflects a broader reluctance to address ergonomic issues openly during training. Additionally, the study revealed that many surgeons, faced with demanding schedules, often rely on self-treatment for physical strain rather than pursuing professional care.

## Conclusion

Collectively, the studies emphasize the multifaceted nature of ergonomic optimization in cleft surgery, highlighting that no single intervention suffices. Personal visualization aids improve precision but may still contribute to MSK strain if improperly used. Emerging technologies like videoscopes, exoscopes, and robotic systems show promise in enhancing both visualization and surgeon comfort, though they often require additional setup, training, and investment. Interventions targeting posture, scheduled micro-breaks, and physical conditioning complement equipment-focused strategies by addressing surgeon well-being holistically.

Nonetheless, limitations persist across the literature, including heterogeneous study designs, small sample sizes, and variability in outcome measures related to MSK symptoms. Additionally, few studies comprehensively evaluate long-term impacts or incorporate standardized ergonomic training, underscoring a critical gap in surgical education.

Future research should prioritize robust, controlled trials comparing ergonomic modalities with standardized outcome metrics and explore the integration of formal ergonomics curricula into surgical training. By continuing to refine and combine ergonomic strategies, the surgical community can better safeguard surgeon health while maintaining high standards of patient care in cleft lip and palate repair.

## Supplemental Material

sj-docx-1-psg-10.1177_22925503251386782 - Supplemental material for Recommendations for Improving Ergonomics in Cleft Palate and Lip Surgery: A Scoping Review Supplemental material, sj-docx-1-psg-10.1177_22925503251386782 for Recommendations for Improving Ergonomics in Cleft Palate and Lip Surgery: A Scoping Review by Sara B. A. Morel, Rawan ElAbd, Youssef ElAbd, Dino Zammit and Sabrina Cugno in Plastic Surgery

sj-docx-2-psg-10.1177_22925503251386782 - Supplemental material for Recommendations for Improving Ergonomics in Cleft Palate and Lip Surgery: A Scoping Review Supplemental material, sj-docx-2-psg-10.1177_22925503251386782 for Recommendations for Improving Ergonomics in Cleft Palate and Lip Surgery: A Scoping Review by Sara B. A. Morel, Rawan ElAbd, Youssef ElAbd, Dino Zammit and Sabrina Cugno in Plastic Surgery
