# Supplementary material for: Recommendations for Improving Ergonomics in Cleft Palate and Lip Surgery: A Scoping Review
Source: Plast Surg (Oakv). 2025 Oct 24:22925503251386782. Online ahead of print. doi: 10.1177/22925503251386782 (PMC12552228; doi:10.1177/22925503251386782)
Supplement: sj-docx-1-psg-10.1177_22925503251386782 - Supplemental material for Recommendations for Improving Ergonomics in Cleft Palate and Lip Surgery: A Scoping Review [file sj-docx-1-psg-10.1177_22925503251386782.docx]

**Supplementary Figure 1**: Search Strategy

**PubMed**

**Search Query Results**

#1 cleft* 61,580

#2 ergonomic* 29,608

#3 “Cleft Palate”[Mesh] 24,214

#4 “Cleft Lip”[Mesh] 18,152

#5 (#1 OR #3 OR #4) AND (#2) 25

**Ovid**

**Search Query Results**

#1 cleft*.mp. 59,368

#2 Cleft Palate/ 24,223

#3 Cleft Lip/ 18,159

#4 Ergonomics/ 21,922

#5 ergonomic*.mp. 13,393

#6 1 OR 2 OR 3 59,368

#7 4 OR 5 21,922

#8 6 AND 7 24

**CINAHL**

**Search Query Results**

#1 cleft* 1,817

#2 MeSH descriptor: [Cleft Palate] explode all trees 514

#3 MeSH descriptor: [Cleft Lip] explode all trees 433

#4 ergonomic* 2,473

#5 MeSH descriptor: [Ergonomics] explode all trees 3,994

#6 ("cleft":ti,ab,kw OR "MeSH descriptor: [Cleft Palate] explode all trees":ti,ab,kw OR "MeSH descriptor: [Cleft Lip] explode all trees":ti,ab,kw) AND ("ergonomic":ti,ab,kw OR "MeSH descriptor: [Ergonomics] explode all trees":ti,ab,kw)

1
