# Supplementary material for: Recommendations for Improving Ergonomics in Cleft Palate and Lip Surgery: A Scoping Review
Source: Plast Surg (Oakv). 2025 Oct 24:22925503251386782. Online ahead of print. doi: 10.1177/22925503251386782 (PMC12552228; doi:10.1177/22925503251386782)
Supplement: sj-docx-2-psg-10.1177_22925503251386782 - Supplemental material for Recommendations for Improving Ergonomics in Cleft Palate and Lip Surgery: A Scoping Review [file sj-docx-2-psg-10.1177_22925503251386782.docx]

**Supplementary Figure 2:** PRISMA Diagram

**Identification of studies via databases and registers**

Records removed *before screening*:

Duplicate records removed (n = 24)

Records identified from*:

PubMed = 25

Ovid = 24

CINAHL = 1

**Identification**

Records screened

(n = 26)

Records excluded**

(n = 10)

Reports sought for retrieval

(n = 16)

**Screening**

Reports assessed for eligibility

(n = 16)

Studies included in review

(n = 0)

**Included**
